# Socioeconomic Status and Race are both Independently associated with Increased Hospitalization Rate among Crohn’s Disease Patients

**DOI:** 10.1038/s41598-018-22429-z

**Published:** 2018-03-05

**Authors:** Caroline Walker, Chaitanya Allamneni, Jordan Orr, Huifeng Yun, Paul Fitzmorris, Fenglong Xie, Talha A. Malik

**Affiliations:** 1 Department of Internal Medicine, Tinsley Harrison Internal Medicine Residency Program, University of Alabama at Birmingham, Birmingham, Alabama USA; 2 Division of Gastroenterology & Hepatology, University of Alabama at Birmingham, Birmingham, Alabama USA; 3 Department of Epidemiology, University of Alabama at Birmingham, Birmingham, Alabama USA

## Abstract

Racial disparities are observed clinically in Crohn’s Disease (CD) with research suggesting African Americans (AA) have worse outcomes than Caucasian Americans (CA). The aim of this study is to assess whether socioeconomic status (SES) rather than race is the major predictor of worse outcomes. We designed a retrospective cohort study of 944 CD patients seen at our center. Patients’ billing zip codes were collected and average income and percent of population living above or below poverty level (PL) for each zip code calculated. Patients were separated by quartiles using average state income level and federal PL. Demographics and hospitalization rates were collected. Poison regression models estimated incidence rate ratios (IRR) for CD-related hospitalizations. Incidence rate (IR) of hospitalization per 100-person years for the lowest income group was 118 (CI 91.4–152.3), highest income group was 29 (CI 21.7–38.9), Above PL was 26.9 (25.9–28.9), Below PL was 35.9 (33.1–38.9), CA was 25.3 (23.7–27), and AA was 51.4 (46.8–56.3). IRR for a CD-related hospitalization for lowest income group was 2.01 (CI 1.34–3.01), for Below PL was 1.26 (CI 1.12–1.42), and for AAs was 1.88 (CI 1.66–2.12). SES and race are both associated with hospitalization among CD patients and need further investigation.

## Introduction

Crohn’s Disease (CD) is a chronic inflammatory disorder affecting approximately 780,000 people in the United States^1^. Transmural inflammation in CD can affect any part of the gastrointestinal tract and is classified as penetrating, structuring, or inflammatory^2–5^. It was initially believed that this disease predominantly affected Caucasians, and it was not until 1966 that inflammatory bowel disease (IBD) was studied in the African American (AA) population^2,6^. Since, a higher incidence of CD has been observed in the general population as well as in AAs^7,8^. In numerous diseases, including cardiac disease, obesity, and diabetes mellitus, AAs suffer poorer outcomes, receive fewer therapeutic interventions, and, in some series, have been found to have higher mortality than their CA counterparts. Our study tests the hypothesis that socioeconomic status (SES) is the main predictor of worse outcomes rather than a biological difference related to race.

## Materials and Methods

### Study Design, Patient Population, and Selection Criteria

We conducted a retrospective cohort study comparing the hospitalization rate for CD among AAs and CAs. We analyzed data from 944 CD patients seen at University of Alabama at Birmingham (UAB), a tertiary IBD center, from 2000 to 2013. Patients were included if they were AA or CA and older than 19 years of age at first diagnosis visit. Last known billing zip codes were collected for each patient and used to obtain US Census data concerning average income and percentage of population living below federal poverty level (FPL) in that zip code. Last billing zip code was assumed to be the same as that at first diagnosis visit. The study was approved by the University of Alabama at Birmingham Office of Institutional Review Board (IRB), and designed and conducted in accordance with the declaration of Helsinki regarding human research. Informed consent was not required for this study, as there were no identifiers of any kind that any participant saw, & no risk to subjects.

### Data Collection and Variable Definitions

#### Social economic status and race

Income, poverty level, and race were all designated as variables of interest. Average income and percentage of population living below the FPL for each zip code were obtained from the United States Census (US Census) 2014 American Community Survey (ACS)^9^. Income level was analyzed in quartiles (QT). Low income (QT L) was defined as zip codes with less than $22000 in income per year. Incomes in this group represent less than 138% federal poverty line (FPL) in 2013 and would qualify for federal and state welfare. Lower middle income (QT LM) was defined as zip codes with median incomes $22000–$43253. This includes those who do not qualify for welfare but who are below the median income for Alabama (AL). Middle income (QT M) was defined as zip codes with median income $43254–$86506. These zip codes include families who make the median income for AL up to 400% of the FPL, the level after which all premium tax credits cease. Upper middle income (QT UM) was defined as zip codes in which the median income is greater than twice the median income for AL (an income >$86506) (Table 1). Poverty Level (PL) of each patient’s home zip area was designated as a variable of interest and analyzed dichotomously. For each patient, we evaluated the percentage of population in that zip code living below the state poverty level. For the state of Alabama, in the average zip code, 18.6% of the population lives below poverty level. Zip codes were classified as being ‘Above PL’ or ‘Below PL’ depending on whether they had greater than or less than 18.6% of the population living below poverty.

**Table 1:** Quartile Definition.

Quartile	Income (US dollars)	Description
Lower class (QT L)	<$22,000	Qualifies for federal and state welfare (including food stamps). Includes those up to 138% of the federal poverty level for a family of two
Lower middle class (QT LM)	$22,000–$43,253	Includes those who do not qualify for welfare but who are below the median income for AL^*^
Middle class (QT M)	$43,254–$86,506	Includes those who make the median income for AL up to 400% of the FPL^*^.The level after which all premium tax credits cease
Upper middle class (QT UM)	>$86,506	Those who make more than twice the median income for AL

^*^AL: Alabama, United States; ^*^FPL: Federal Poverty Level.

### Outcome of interest

As the outcome of interest, A CD-related hospitalization was defined as any hospital admission for a CD complication including infections, fistula, strictures, or exacerbations.

### Other covariates

Data collected by review of electronic medical records (EMR) at the time of first observation included age, sex, tobacco use, duration of CD (DOD), metabolic syndrome, and exposure to steroids, thiopurines, methotrexate, and biologics. Last billing zip code was assumed to be the same as that at first diagnosis visit. Steroid use was defined as exposure to oral or parenteral corticosteroids during the period of observation for at least four weeks. Thiopurine use was defined as use of azathioprine (AZA) or 6-mercaptopurine (6-MP) for at least four weeks during the period of observation. Methotrexate (MTX) use was defined as use of MTX for at least four weeks during the period of observation. Biologic use was defined as use of any biologic agent for at least four weeks during the period of observation. Patients were considered to have metabolic syndrome if they had the diagnosis of at least three of the following at first observation: hypertension, diabetes mellitus, hypertriglyceridemia, low HDL levels, or obesity (ATP III criteria). For each patient, the period of observation was defined as the time in years between the first and the last documented encounter at our tertiary care center during the years 2000 through 2013.

### Statistical Analysis

For each patient, the follow-up time started from the first documented encounter at our tertiary care center and ended at the last visit during the years 2000 through 2013. For each variable of interest (QT, PL, and race) median age, duration of disease (DOD), and proportion of women, AAs, patients who met criteria for metabolic syndrome, and tobacco users were calculated. Percent of patients exposed to steroids, thiopurines, methotrexate, and biologics were also calculated.

Hospitalization rates and 95% confidence intervals for each of the variables of interest were calculated. Poisson regression models were used to estimate incidence rate ratios (IRR) and 95% confidence intervals for CD-related hospitalizations. In addition to the crude model, a second model was used which adjusted for age, sex, race, tobacco use, metabolic syndrome, DOD, and drug exposure (steroids, thiopurines, methotrexate, and biologics). All statistical analyses were conducted using SAS 9.4. Statistical tests were two-sided with a significance level alpha <0.05. The datasets generated during and/or analyzed during the current study are available from the corresponding author on reasonable request.

## Results

### Income

Of the 944 patients observed, there were 13 patients in QT L, 403 patients in QT LM, 497 patients in QT M, and 31 patients in QT UM. Mean age was similar across all groups with a range of 39–45 years. Mean period of observation was 4.7 ± 3.6 years, and median period of observation was 4 years. For all groups, except QT L, women made up the majority of the observed population with 58–66% of observed patients. Groups’ minority composition varied inversely with income. QT UM was comprised of only 9.7% AA patients whereas QT L was nearly 54% AA. 23% of QT L patients used tobacco and 3% of QT UM patients used tobacco. Presence of metabolic syndrome was similar across groups with the exception of QT L where no patients were found to have metabolic syndrome. Biologic exposure was similar across all income quartiles. QT L had both greater exposure to thiopurine (69.2%) and lower exposure to steroids (30.7%) than other groups. Otherwise, exposure was similar in the other groups. Detailed results appear in Table 2. Hospitalization rate was highest in QT L with nearly 118 hospitalizations per 100-person years (CI 91.4–152.3). Hospitalization rate in QT LM was 32.2 per 100-person years (CI 29.7–34.8), QT M 26.8 (CI 24.7–29), and QT UM 29 (CI 21.7–38.9).

**Table 2:** Income as the Variable of Interest.

Characteristics	Quartile			
	QT L N = 13	QT LM N = 403	QT M N = 497	QT UM N = 31
Mean age, y (SD)	39.8 (10.21)	45.2 (16)	42.3 (15.9)	45.2 (18)
Mean DOD^*^, y (SD)	10.6 (6.8)	13.7 (10.5)	13.2 (11.1)	16.4 (10.8)
Male (%)	53.8	34.2	38.2	41.9
Race (%)				
Caucasian	46.2	68.7	79.9	90.3
African	53.8	29.5	18.3	9.7
American				
Other	0	1.7	1.8	0.0
Tobacco Use (%)	23.1	27.3	19.1	3.2
Metabolic Syndrome (%)	0	5.5	3.9	3.9
Steroid Use (%)	30.8	57.6	51.7	54.8
Thiopurine Use (%)	69.2	51.1	52.9	61.3
Biologic Use (%)	46.2	52.6	51.9	48.4
Above PL^*^ (%)	0	20.6	94.3	100
Median Income, US dollars (IQR^*^)	19564 (18894, 20894)	34583 (31100, 38821)	54852 (48865, 71871)	95196 (94504, 120943)
PL, median (IQR)	40.6 (38.9, 42.5)	24.0 (19.6, 28.3)	10.5 (6.6, 15.8)	4.8 (2.7, 5.4)

^*^DOD: Duration of Disease; ^*^PL: Poverty Level; ^*^IQR: Interquartile Range (1^st^, 3^rd^ quartiles).

### Poverty Level

Of the 944 patients observed, US Census data on PL data was available for 941 patients. The majority of the patients observed (62.1%) lived in zip codes where <18.6% of the residents are living below the poverty level (Above PL). The Above PL and Below PL groups had similar mean ages, duration of disease, and sex compositions. Exposure to steroids, thiopurines, and biologics as well as incidence of metabolic syndrome was similar. A greater proportion of tobacco use was seen in the Below PL group. The Below PL was 34.6% AA while the Above PL was 16.1% AA. Table 3 displays the demographics of poverty as the variable of interest. Hospitalization rate in the Below PL group was 35.9 per 100-person years (CI 33.1–38.9) and 26.9 per 100-person years (CI 25.9–28.9) in the Above PL group).

**Table 3:** Poverty Level as the Variable of Interest.

Characteristics	Poverty Level	
	Above PL N = 585	Below PL N = 356
Mean age, y (SD)	43.2 (16.3)	44.3 (15.6)
Mean DOD^*^, y (SD)	13.8 (11.1)	13 (10.2)
Male (%)	38.1	35.1
Race (%)		
Caucasian	82.4	63.5
African American	16.1	34.6
Other	1.5	2
Tobacco Use (%)	19.8	26.1
Metabolic Syndrome (%)	3.7	5.6
Steroid Use (%)	52.3	57.6
Thiopurine Use (%)	52.7	52.8
Biologic Use (%)	52.7	50.8
Median Income, US dollars (IQR^*^)	53773 (46258, 71904)	33533 (30230, 38694)
PL^*^, median (IQR)	10.5 (6.3, 15.8)	26.0 (22.2, 29.6)

^*^DOD: Duration of Disease; ^*^PL: Poverty Level; ^*^IQR: Interquartile Range (1^st^, 3^rd^ quartiles).

### Race

As seen in Table 4, mean ages, tobacco use, duration of disease, drug exposure, and presence of metabolic syndrome were similar for AA and CA. The AA group contained a slightly higher proportion of females (70.9% vs 61.5%). The AA patient was more likely to live in a zip code where greater than 18.6% of the population lived below FPL (55.9% vs 31.8%). Hospitalization rates per 100-person years for AA was 51.4 (CI 46.8–56.3) and CA was 25.3 (CI 23.7–27) (Table 5).

**Table 4:** Race as the Variable of Interest.

Characteristics	Race	
	Caucasian N = 711	African American N = 220
Mean age, y (SD)	44.6 (16.5)	40.9 (13.6)
Mean DOD^*^, y (SD)	14.4 (11.2)	11 (8.5)
Male (%)	38.5	29.1
Tobacco Use (%)	22.1	23.6
Metabolic Syndrome (%)	4.4	5.1
Steroid Use (%)	53.7	55.5
Thiopurine Use (%)	53.3	50.9
Biologic Use (%)	52.3	51.8
Below PL^*^ (%)	31.8	55.9

^*^DOD: Duration of Disease; ^*^PL: Poverty Level.

**Table 5:** Incidence Rates and Incidence Rate Ratios (IRR) of Crohn’s Disease Related Hospitalizations.

	CD-related number of hospitalizations/100-person years	Hospitalization Rate (95% CI)^*^	IRR (95% CI)	
			Model 1	Model 2
Income Quartile				
QT L	50/59	118 (91.4–152.3)	4.06 (2.76–5.99)	2.01 (1.34–3.01)
QT LM	610/1896	32.2 (29.7–34.8)	1.11 (0.82–1.5)	0.67 (0.49–0.92)
QT M	612/2288	26.8 (24.7–29)	0.92 (0.68–1.25)	0.57 (0.42–0.77)
QT UM	45/155	29 (21.7–38.9)	1 (reference)	1 (reference)
Above PL^*^	732/2726	26.9 (25.9–28.9)	1 (reference)	1 (reference)
Below PL	599/1669	35.9 (33.1–38.9)	1.34 (1.20–1.49)	1.26 (1.12–1.42)
Race				
Caucasian	874/3455	25.3 (23.7–27)	1 (reference)	1 (reference)
African American	454/884	51.4 (46.8–56.3)	2.03 (1.81–2.27)	1.88 (1.66–2.12)

^*^Per 100 person-years; ^*^PL: Poverty Level.

Model 1 is unadjusted.

Model 2 is adjusted for age, sex, tobacco use, duration of disease (DOD), metabolic syndrome, steroid use, thiopurine use, and biologic use. Model 2 is also adjusted for race in income quartile and Below PL variables.

### Rate Ratios

Rate ratios are summarized in Table 5. Those in the lowest income group were hospitalized at a rate four times greater than that of the highest income group in the unadjusted model (IRR QT D 4.06; CI 2.76–5.99) and two times greater in the adjusted model (IRR 2.01; CI 1.34–3.01). There was no significant difference in the rates of hospitalization for those in QT LM and QT M in the crude model (Model 1). Once adjusted for age, sex, race, tobacco use, duration of disease, metabolic syndrome, and drug exposure QT LM and QT M both reached significance (IRR QT LM 0.67; CI 0.49–0.92, IRR QT M 0.57; CI 0.42–0.77) as compared to QT UM and were less likely to be admitted than those in QT UM. Crude models demonstrated that patients in the Below PL group were hospitalized at a rate of 1.34 (CI 1.20–1.49) times more than those in the Above PL group. This remained significant in Model 2 (IRR 1.26; CI 1.12–1.42). Crude analysis of rate ratios demonstrates that AAs were 2.03 (CI 1.81–2.27) times more likely to be hospitalized for a CD-related event than CA. This remains true in the adjusted model with an IRR of 1.88 (CI 1.66–2.12).

## Discussion

Results from this retrospective cohort study confirm that race and SES are both associated with the clinical course of CD. We observed that AAs were more likely to be hospitalized for a CD-related event than CAs. This remained true even when adjusted for age, sex, tobacco use, duration of disease, metabolic syndrome, drug exposure, and SES. These results are in line with prior research from our group concluding that AAs had high rate of CD-related hospitalizations than CAs^10^. Analysis by both income and poverty level (PL) revealed a trend of decreasing hospitalization rate with increasing income level. Interestingly, those in QT LM and QT M appeared to have lower rates of hospitalization than those in QT UM in the adjusted model. This could be a falsely elevated rate due to the relatively small sample size in QT UM, but could also represent higher utilization of health care compared to middle and lower middle-income patients.

Analysis by poverty level was consistent with the trend seen with median income, in that those in areas with greater than 18.6% living ‘Below PL’ were more likely to be hospitalized for a CD-related event. Demographics of each variable of interest show that AAs comprised the majority of the low-income quartile (53.8%) and a greater proportion of the Below PL group (34.6%) (Tables 2, 3). Also, when race was designated as the variable of interest, the majority of AA analyzed in this study lived in areas designated Below PL (55.9%) (Table 4).

The complexity of CD is further enhanced by the racial and SES differences observed in clinical practice. In 1986, Goldman *et al*. concluded that CD was less common in AAs, though noted that CD was more aggressive with an earlier age of onset and development of more complications over time in AAs^11^. Other studies have also demonstrated that AAs experience more CD complications, in particular rheumatologic manifestations^12^. The role of race in CD cannot be fully understood without appreciating the contributions of socioeconomic status. Straus *et al*. concluded that disparities in disease severity were in fact a result of social and economic inequalities, including affordability of healthcare, and difficulties with travel to a provider’s office, rather than genetic differences^13^. These results have been confirmed by other studies and metaanalyses^14^.

Our study shows the importance of accounting for socioeconomic factors when examining the racial or ethnic differences in CD. Analysis by income and poverty level displayed the trend that patients of greater income are less hospitalized for a CD-related event. Socioeconomic factors are most frequently implicated in the disparities seen in US health populations^15,16^. For example, people of lower SES are less likely to obtain needed transportation for disease management. Wallace *et al*. estimated that 3.6 million people do not obtain medical care yearly due to transportation barriers^17^. Those affected were more likely to be older, poorer, less educated, and of a racial or ethnic minority. Surveys have also demonstrated that AAs have higher travel burdens as compared to CAs, even when controlling for SES^18^. Guidry *et al*. also confirmed that AAs had greater barriers to transportation than CAs including distance to treatment, access to vehicles, and difficulty finding a driver^19^. Ability to afford health insurance is yet another barrier to care. Those who are uninsured are seven times more likely to forgo needed health care, with the burden falling most heavily on the young and members of minority groups^20^. One in four adults aged 19–64 report not having health insurance at some point during 2011, with the majority remaining uninsured for greater than one year^20^. The percentage of non-Hispanic blacks who are uninsured is significantly higher than Caucasians (26.2 vs 16.1 in the year 2010, p = 0.001). Studies demonstrate that low-income minorities have poorer outcomes than their privately insured counterparts^21,22^. Access to health care and insurance are particularly important in CD which requires frequent follow-up, laboratory monitoring, endoscopic procedures, and often monthly infusions. Those with greater financial resources, regular employment, and advanced education may be more likely to have the means that promote superior health outcomes^23^.

Results show that both people of lower SES and AAs independently have worse CD outcomes with regards to hospitalization which begs the question if SES is the greater predictor of worse outcomes rather than a biological difference related to race. Furthermore, while results show that people of lower SES had an increased rate of CD-related hospitalization, demographic data revealed that the majority of these patients were AA. Results also show that AAs had an increased rate of CD-related hospitalization and the majority of these patients lived in areas of Below PL. It can be reasonably concluded that the predominant driver of this association may indeed be lower SES among AA patients but it does not entirely explain it. As such, we draw significant correlation between these two independent variables to show that race and SES are closely related.

One of the main limitations of our study is the small population size in QT L. A larger population for this QT would allow for direct analysis and measurement of outcomes of all racial groups in a low-income QT. The retrospective nature of the study is also a limitation. Another limitation in our study is that our results were found using income data for the state of Alabama and may not represent other areas of the country. In addition, the assumption was made that individual zip codes accurately reflected income of studied individuals, while greater variability may have existed. Also, as a tertiary care referral center, it is plausible that a large proportion of patients seen represents lower SES and receive charity care, thus skewing the data towards poorer outcomes due to lower SES. Analyzing SES by income and poverty level allows for greater generalizability of our findings however similar results may not be reproduced at a smaller, private health center. Finally, our study failed to evaluate prolonged adherence to CD drug therapy, as we assessed exposure to steroids, thiopurines, methotrexate, and biologics for only at least four weeks during the observation period. Thus, it is plausible that patients of lower SES may have been less compliant with treatment over time, which modified their disease course and hospitalization rate. However, the median duration of observation of each individual patient was four years, suggesting adherence was likely less of an issue.

Further investigation to better understand racial differences in CD is warranted, and should directly compare outcomes in both AAs and CAs in the same SES group as well as AAs of different SES groups.

## Conclusions

This study was intended to examine the relationship between SES and race as predictors of outcome in CD. Our results suggest that patients of lower SES, as defined by lower incomes and living in areas of Below PL, have significantly increased rates of CD-related hospitalization. Our results also show that AAs have significantly increased rates of hospitalization for CD-related events. All variables of interest (income, poverty level, and race) show that more AAs comprise lower income quartiles and live in areas of Below PL further supporting the significance of these two independent variables and suggesting an underlying correlation. While SES is a predominant driver of poorer outcomes in Crohn’s among AA patients, it does not entirely explain it. Further studies are needed to support these conclusions. In addition to complex inflammatory pathophysiology, difficult medical management, and significant morbidity and mortality CD is further complicated by racial and SES disparities. It is imperative to understand and address these differences to better provide care for CD patients in the future.
